# Supplementary material for: Smooth Interpolating Curves with Local Control and Monotone Alternating Curvature
Source: Comput Graph Forum. 2022 Oct 6;41(5):25–38. doi: 10.1111/cgf.14600 (PMC9827861; doi:10.1111/cgf.14600)
Supplement: Supplementary file 1 — Supplement Material [file CGF-41-25-s001.zip › Local-Smooth-Interpolating-MonoCurvature/extern/clothoids/submodules/Utils/docs/genindex.html]

Index — Utils v1.2

### Navigation

- index
- toc
- Utils »
- Index

# Index

**C**
| **D**
| **H**
| **I**
| **R**

## C

- cpuInfo (function)

## D

|  |  |
| --- | --- |
| - detail::basic\_data (C++ struct) | - detail::format\_float (function) |

## H

|  |  |
| --- | --- |
| - has\_3Dnow (function) - has\_3DnowExt (function) - has\_MMX (function) - has\_MMXplus (function) - has\_SSE (function) - has\_SSE2 (function) | - has\_SSE3 (function) - has\_SSE41 (function) - has\_SSE42 (function) - has\_SSE4a (function) - has\_SSE5 (function) - has\_SSSE3 (function) |

## I

- info (function)

## R

|  |  |
| --- | --- |
| - rang::bg (C++ enum) - rang::bg::black (C++ enumerator) - rang::bg::blue (C++ enumerator) - rang::bg::cyan (C++ enumerator) - rang::bg::gray (C++ enumerator) - rang::bg::green (C++ enumerator) - rang::bg::magenta (C++ enumerator) - rang::bg::red (C++ enumerator) - rang::bg::reset (C++ enumerator) - rang::bg::yellow (C++ enumerator) - rang::bgB (C++ enum) - rang::bgB::black (C++ enumerator) - rang::bgB::blue (C++ enumerator) - rang::bgB::cyan (C++ enumerator) - rang::bgB::gray (C++ enumerator) - rang::bgB::green (C++ enumerator) - rang::bgB::magenta (C++ enumerator) - rang::bgB::red (C++ enumerator) - rang::bgB::yellow (C++ enumerator) - rang::control (C++ enum) - rang::control::Auto (C++ enumerator) - rang::control::Force (C++ enumerator) - rang::control::Off (C++ enumerator) - rang::fg (C++ enum) - rang::fg::black (C++ enumerator) - rang::fg::blue (C++ enumerator) - rang::fg::cyan (C++ enumerator) - rang::fg::gray (C++ enumerator) | - rang::fg::green (C++ enumerator) - rang::fg::magenta (C++ enumerator) - rang::fg::red (C++ enumerator) - rang::fg::reset (C++ enumerator) - rang::fg::yellow (C++ enumerator) - rang::fgB (C++ enum) - rang::fgB::black (C++ enumerator) - rang::fgB::blue (C++ enumerator) - rang::fgB::cyan (C++ enumerator) - rang::fgB::gray (C++ enumerator) - rang::fgB::green (C++ enumerator) - rang::fgB::magenta (C++ enumerator) - rang::fgB::red (C++ enumerator) - rang::fgB::yellow (C++ enumerator) - rang::style (C++ enum) - rang::style::blink (C++ enumerator) - rang::style::bold (C++ enumerator) - rang::style::conceal (C++ enumerator) - rang::style::crossed (C++ enumerator) - rang::style::dim (C++ enumerator) - rang::style::italic (C++ enumerator) - rang::style::rblink (C++ enumerator) - rang::style::reset (C++ enumerator) - rang::style::reversed (C++ enumerator) - rang::style::underline (C++ enumerator) - rang::winTerm (C++ enum) - rang::winTerm::Ansi (C++ enumerator) - rang::winTerm::Auto (C++ enumerator) - rang::winTerm::Native (C++ enumerator) |

### Quick search

### Table of Contents

- rang - Colors for your Terminal
- Zstr
- C++ API

«
hide menu

menu
sidebar
»

### Navigation

- index
- toc
- Utils »
- Index

© Copyright 2021, Enrico Bertolazzi.
Created using Sphinx 4.2.0.
